# GLRX5-associated [Fe-S] cluster biogenesis disorder: further characterisation of the neurological phenotype and long-term outcome

**DOI:** 10.1186/s13023-021-02073-z

**Published:** 2021-11-03

**Authors:** Bindu Parayil Sankaran, Sachin Gupta, Michel Tchan, Beena Devanapalli, Yusof Rahman, Peter Procopis, Kaustuv Bhattacharya

**Affiliations:** 1 Department of Biochemical Genetics and Genetic Metabolic Disorders Service, The Children’s Hospital at Westmead, Westmead, NSW Australia; 2 The Children’s Hospital at Westmead Clinical School, Faculty of Medicine and Health, University of Sydney, Sydney, NSW Australia; 3 T.Y Nelson Department of Neurology, The Children’s Hospital at Westmead, Westmead, NSW Australia; 4 Westmead Hospital, Faculty of Medicine and Health, University of Sydney, Sydney, Australia

**Keywords:** GLRX5, Fe-S cluster biogenesis, Non ketotic hyperglycinemia, Spastic paraplegia, SPON, Lipoic acid, Sodium benzoate

## Abstract

**Background:**

Identification and characterisation of monogenic causes of complex neurological phenotypes are important for genetic counselling and prognostication. Bi-allelic pathogenic variants in the gene encoding GLRX5, a protein involved in the early steps of Fe-S cluster biogenesis, are rare and cause two distinct phenotypes: isolated sideroblastic anemia and a neurological phenotype with variant non-ketotic hyperglycinemia. In this study, we analysed the evolution of clinical and MRI findings and long-term outcome of patients with *GLRX5* mutations.

**Methods:**

Four patients from three Australian families of Lebanese descent were identified. All patients presented in childhood and were followed up into adult life through multiple clinical assessments. All were prescribed sodium benzoate.

**Results:**

All patients (all females, age range 18–56 years) showed a complex neurological phenotype characterised by varying combinations of spastic paraparesis, length-dependent motor/sensory-motor axonal polyneuropathy, and psychiatric disturbances with variable intellectual disability. All had non-ketotic hyperglycinemia and a homozygous pathogenic c.151_153delAAG (p.K51del) change in *GLRX5.* Motor disability gradually progressed reaching moderate disability during adolescence and moderately severe disability during adult life. The major MRI finding was the upper cervical spinal cord signal changes with contrast enhancement noted in all and additional leukoencephalopathy in one. On follow up MRI, the white matter lesions diminished on a subsequent scan and then remained static over time. The spinal cord showed gliotic changes. Two patients have previously demonstrated low pyruvate dehydrogenase complex deficiency but none had plasma lactate elevation, nor biochemical evidence of branch-chain keto-dehydrogenase deficiency. Glycine levels reduced in patients that tolerated sodium benzoate, possibly stabilising clinical manifestations.

**Conclusions:**

This report demonstrates that the p.K51del *GLRX5* variant causes a distinct and predictable neurological phenotype. The clinical assessments spanning from childhood to adult life enable physicians to infer the natural history of GLRX5 related neurological disorder. There may be widespread metabolic consequences, and optimal management is unknown.

## Introduction

Glutaredoxin5 (GLRX5) is a 156 amino acid mitochondrial protein, involved in iron-sulfur [Fe-S] cluster biogenesis [1]. Fe-S clusters are ancient and ubiquitous classes of cofactors that are essential for many fundamental biological processes which range from electron transport to DNA repair [1–3]. Mitochondria play the central role in Fe-S cluster biogenesis through a highly conserved Fe-S cluster assembly machinery (ISC machinery) which also controls the synthesis of nuclear and cytosolic Fe-S proteins. The molecular mechanisms involved in mitochondrial [Fe-S] biogenesis and maturation and their role in human diseases have been an area of intense research during the last two decades [1]. It has been shown that defects in this highly complex process lead to severe neurological, hematological and multi-systemic diseases [4–6].

GLRX5 is involved in the early steps in Fe-S biogenesis and is considered to play a role in the transfer of Fe-S cluster from the scaffold to target proteins, the second step in Fe-S biogenesis [1]. Mutations in *GLXR5* have been associated with two distinct phenotypes so far, variant non ketotic hyperglycinemia and isolated sideroblastic anemia [7, 8]. Isolated sideroblastic anemia was first reported in a single patient with a GLRX5 messenger RNA splcing defect [7]. Our group described the clinical presentation of one patient in 2007 and subsequently another patient presenting with spastic diplegia was identified [9]. These two patients and a third with a similar phenotype led to the identification GLRX5 variant non ketotic hyperglycinemia as described by Baker et al. [8, 10]. Functional analyses of the variant in that study confirmed its deleterious impact on Fe-S cluster biogenesis and subsequent failure of lipoate synthesis with associated deficiency in pyruvate dehydrogenase complex. Two patients with neurological phenotypes have been reported since then and one patient with sideroblastic anemia reported in association with missense mutations in *GLRX5 *[11–13].

This report aims to characterise the neurological phenotype of *GLRX5* over the course of childhood into adult life in four patients emphasising the long term follow up, management and neurological outcome. We demonstrate that the *GLRX5* mutations cause a distinct and predictable neurological phenotype akin to SPOAN (Spastic paraplegia optic atrophy neuropathy, OMIM #609,541). The clinical assessments and multiple evaluations spanning from childhood to adult life enable an inference of the natural history of this disorder.

## Patients and methods

These four patients were identified and continued care from the Genetic Metabolic Disorders database of both children and adult services at Westmead in Sydney Australia. The details of the biochemical and genetic evaluation and limited clinical findings of the first two patients have already been described [8, 9]. *GLRX5* sequencing was undertaken in patient 3 and 4 given the similar clinical and biochemical phenotype to the first 2 patients. All patients had the same pathogenic p.K51del in *GLRX5.* All four patients originated from Kfarsghab in Lebanon. The study was approved by the institute ethics committee and all the patients gave written informed consent. The clinical features of the four patients are summarised in Table 1 and detailed descriptions of the clinical course is as below.

**Table 1:** Clinical presentation and features of four patients with homozygous mutation c.151_153delAAG of GRXL5

Clinical data	Patient 1	Patient 2	Patient 3	Patient 4
Age of onset	2.5 years	6 years	3 years	6 years
Age glycine noted	2.5 years	8 years	32 years	21 years
Presenting features	Lower limb weakness leading to spastic diplegia	Unsteady gait with frequent falls leading to spastic diplegia	Gait difficulty after febrile seizure at 3 years. Walking frame used by 11 years. Wheelchair bound by 30 years	Frequent falls aged 6 years. Decreased ambulation to 40 m by 13 years. Seizure from age 15 years
Baseline plasma glycine (µmol/L)	844–1249 (119–368)	804 (119–368)	586–705 (119–368)	730–1224 (119–368)
CSF glycine (µmol/L)	20–26 (3–9)	15 (3–9)	28 (3–9)	53 (3–9)
CSF/Plasma ratio	0.02–0.03 (< 0.05)	0.02 (< 0.05)	0.04 (< 0.05)	0.04 (< 0.05)
Axonal sensory neuropathy	Identified age 14 years	Identified aged 10 years	Identified after age 30 years when assessed	Identified when assessed after 21 years of age
Age at last review	20 years	19 years	56 years	45 years
Clinical features	Spastic quadriparesis with lower limb diplegia. Mainly ambulatory with wheelchair	Episodes of disabling neuropathic pain after exercise. Spastic diplegia	Mainly ambulant with wheelchair. Obsessive compulsive disorder	Wheelchair dependent. Visual impairment. Mild intellectual impairment
Rankin score	3	3	3	3
Reflex sympathetic dystrophy	Mild bluish discolouration of the feet	Mild bluish discolouration of the feet	Extensive in lower limbs Oedema, hyperesthesia, skin colour changes. Toe amputations	Extensive in lower limbs Oedema, hyperesthesia, skin colour changes. No venous insufficiency
Peripheral nerve/electrophysiology	Impaired vibration and joint position sense in the lower limb. Absent CMAP in CP and posterior tibial	impaired vibration and joint position sense in the lower limb. Absent	Reduced sensation, impaired vibration and joint position sense in the lower limb absent Ankle jerks	Reduced sensation, impaired vibration and joint position sense in the lower limb. Absent Ankle jerks. Absent sensory/motor AP lower limbs-present upper limbs
Fundoscopy	Normal	Mild pallor of the discs	Bilateral Optic disc pallor	Severe disc pallor-atrophy
Cognition	Age appropriate basic language. Difficulties with complex high level language processing tasks, executive and attention problems	Academically gifted attending undergraduate university Bachelor of Science	Normal neuropsychometric test age 47 years	Did not finish school. Aged 39 years—neuropsychometric test—borderline low intellectual function
MRI brain	Persistent white matter signal changes	Mild focal white matter signal changes at 9 years improved by 15 years	Normal at age 49 years	Normal at age 38 years

Modified Rankin scale of disability (0–6 with 0 being unaffected – 3 corresponds to moderate disability; requiring some external help but able to walk without the assistance of another individual

### Patient 1

Now aged 20 years was the second born child to non-consanguineous Australian parents of Lebanese origin originally published by Chiong et al. 2007 and subsequently by Baker et al. [8. She was born after a normal pregnancy and delivery. Her early developmental milestones were normal with supportive speech therapy. She presented at the age of 2.5 years, with a sudden onset of gait difficulty following a mild injury. Neurologic examination showed exaggerated deep tendon reflexes, bilateral ankle clonus and extensor plantar responses without any sensory signs or sphincter dysfunction. Cranial nerve and ophthalmologic examinations were normal. Over the next few months, there was deterioration in her gait with increased spasticity in her lower limbs. There was no regression in cognitive skills or speech. She had two more acute deteriorations associated with intercurrent illnesses in the first year with improvement to near baseline.

At the age of 7 years, there were concerns regarding reduced visual acuity, 6/18 on both eyes, and an ophthalmologist found mildly pale optic discs. There was no further deterioration in her visual acuity. At 14 years, she was able to walk with crutches. She has mild learning difficulties with age-appropriate mathematics and reading skills but poor concentration. Her neurologic signs are static, with spastic diplegia and increased tone in the upper and lower extremities. Nerve conduction studies at the age of 14 years revealed absent compound muscle action potentials in the lower limbs suggesting a length-dependent motor axonal neuropathy. Clinical findings at age 20 years are indicated in Table 1.

#### Investigations

**Fig. 1:**
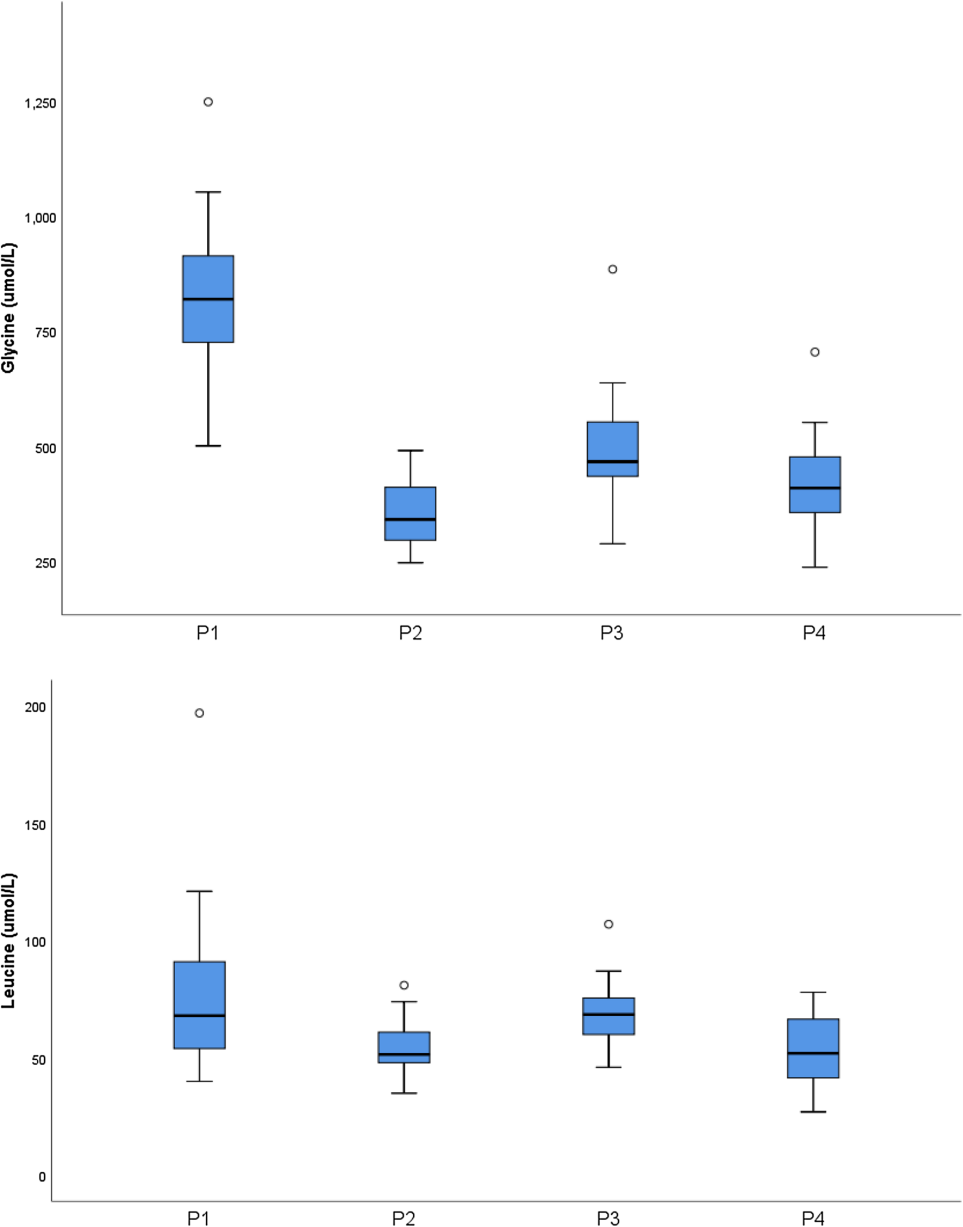
Box plot indicating median and interquartile ranges in box, (and full range with lines) of plasma glycine and leucine levels for the span of treatment, after diagnosis of variant NKH had been established in four patients. P1—Monitored from 4 to 17 years (18 tests). P2—Monitored from 8.5 to 19 years (41 tests). P3—Monitored from 33 to 57 years (30 tests). P4—Monitored from 22 to 46 years (45 tests)

At presentation, she underwent extensive neurometabolic investigations including elevations in glycine in plasma and CSF (Table 1). Her glycine levels were monitored throughout her clinical course as indicated in Fig. 1. Glycine cleavage enzyme analysis was performed on a liver sample, which showed low activity of the P-protein as previously reported [9].

**Fig. 2:**
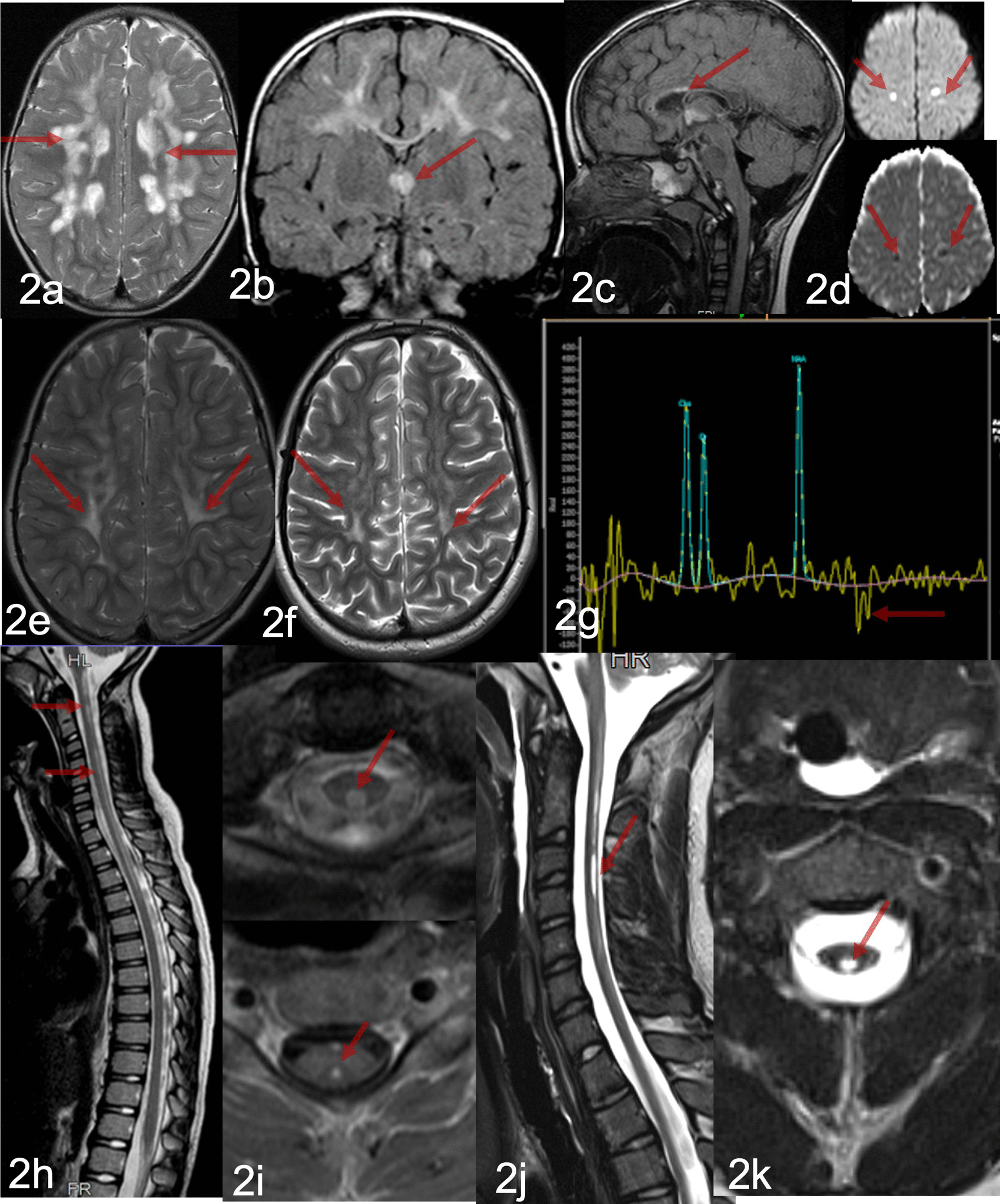
Serial MRI Brain and Spine in Patient 1. Brain magnetic resonance imaging (MRI) at the age of 2½ years (**a**—T2 weighted axial view) shows hyperintense confluent signal changes in the periventricular and deep white matter (**a**). MRI obtained 10 months after presentation showed additional lesions in thalamus and corpus callosum (**b**, **c** arrows). Diffusion-weighted imaging (**d** upper panel and corresponding ADC maps, Fig 1d lower panel) showed restricted diffusion of few white matter lesions (arrows)). **e** T2 weighted axial view: At the age of 5 years MRI showed attenuation of the white matter lesions MR examination at the age of 17 years the white matter lesions were unchanged (**f**, T2 weighted axial). **g** MRS showed lactate peak on multiple occasions. **h** T2w sagittal: MRI spine at the age of 5 years shows spinal cord lesions in the dorsal and central regions extending from the cervico-medullary junction down to T4). The spinal cord lesions showed enhancement with contrast (**i**—T1 w axial image, upper panel, and (lower panel, contrast enhanced image). The spinal cord lesions showed gliotic changes (**j**, T2 weigted sagittal and **k**, T2 weighted axial)

Cranial magnetic resonance imaging (MRI) at the age of 2½ years revealed T2/FLAIR hyperintense confluent signal changes in the periventricular and deep white matter more prominent in the frontal and parietal white matter, with relative sparing of the subcortical U fibres and focal areas of cystic change (Fig. 2a). Spinal MRI was normal She underwent multiple MR imaging examinations during the course of the illness. During the first year, there was a mild increase in MRI lesions with new focal lesions in the brain stem, corpus callosum, temporal lobes, thalamic regions(Fig. 2b, c), and occipital lobes. There was no contrast enhancement of white matter lesions. Diffusion-weighted imaging obtained 10 months after presentation showed restricted diffusion (Fig. 2d). The brain MRI obtained at the age of 5 years showed attenuation of the white matter lesions (Fig. 2e) MR examination at the age of 17 years showed that the white matter lesions were unchanged (Fig. 2f) MRS (Fig. 2g) showed lactate peak on multiple occasions. MRI spine at the age of 5 years revealed spinal cord lesions in the dorsal and central regions extending from the cervico-medullary junction down to T4 (Fig. 2h). The spinal cord lesions showed enhancement with contrast (Fig. 2h, i ). The spinal cord lesions showed gliotic changes (Fig. 2j, k) None of the follow-up spinal MR examinations showed contrast enhancement or restricted diffusion. Her EEG at the same time was normal.

#### Management

She received sodium benzoate and a protein-restricted diet for a few years but was non-compliant afterwards due to fear of swallowing tablets and normalised protein intake from ten years of age. She has also received botulinum toxin injections every three months for lower-limb spasticity and appropriate rehabilitation services. A therapeutic trial of 300 mg α-lipoic acid twice daily, in the absence of sodium benzoate for one year did not have an effect on neurology or plasma glycine, with baseline glycine being 867 µmol/L and two glycine levels being 968 and 890 µmol/L on lipoic acid treatment.

### Patient 2

The second identified case is aged 19 years and was briefly summarised by Baker et al. [8. She was born full-term through an uncomplicated pregnancy and delivery. Her developmental milestones were age-appropriate. She presented at the age of 8 years with a two-year history of flat feet and unsteadiness. Over the prior few months she had been falling over frequently. There was no history of cognitive decline, issues with vision, or bladder and bowel dysfunction. On examination, she had pyramidal tract signs in the lower limbs with normal cranial nerves and upper limbs. The optic discs appeared normal. Vibration was impaired in both legs with preserved proprioception, pain and temperature sensations.

Neurometabolic work up showed significantly elevated urinary, plasma and CSF glycine. The ratio of CSF: plasma glycine ratio was normal at 0.018 (Table 1). A nerve conduction study showed motor axonal neuropathy in the lower limbs. Sodium benzoate was commenced at a dose of 250 mg/kg/day and has been maintained. She has never had a protein restricted diet. Clinically, there was little change in her gait, but there were no further episodes of acute paresis.

**Fig. 3:**
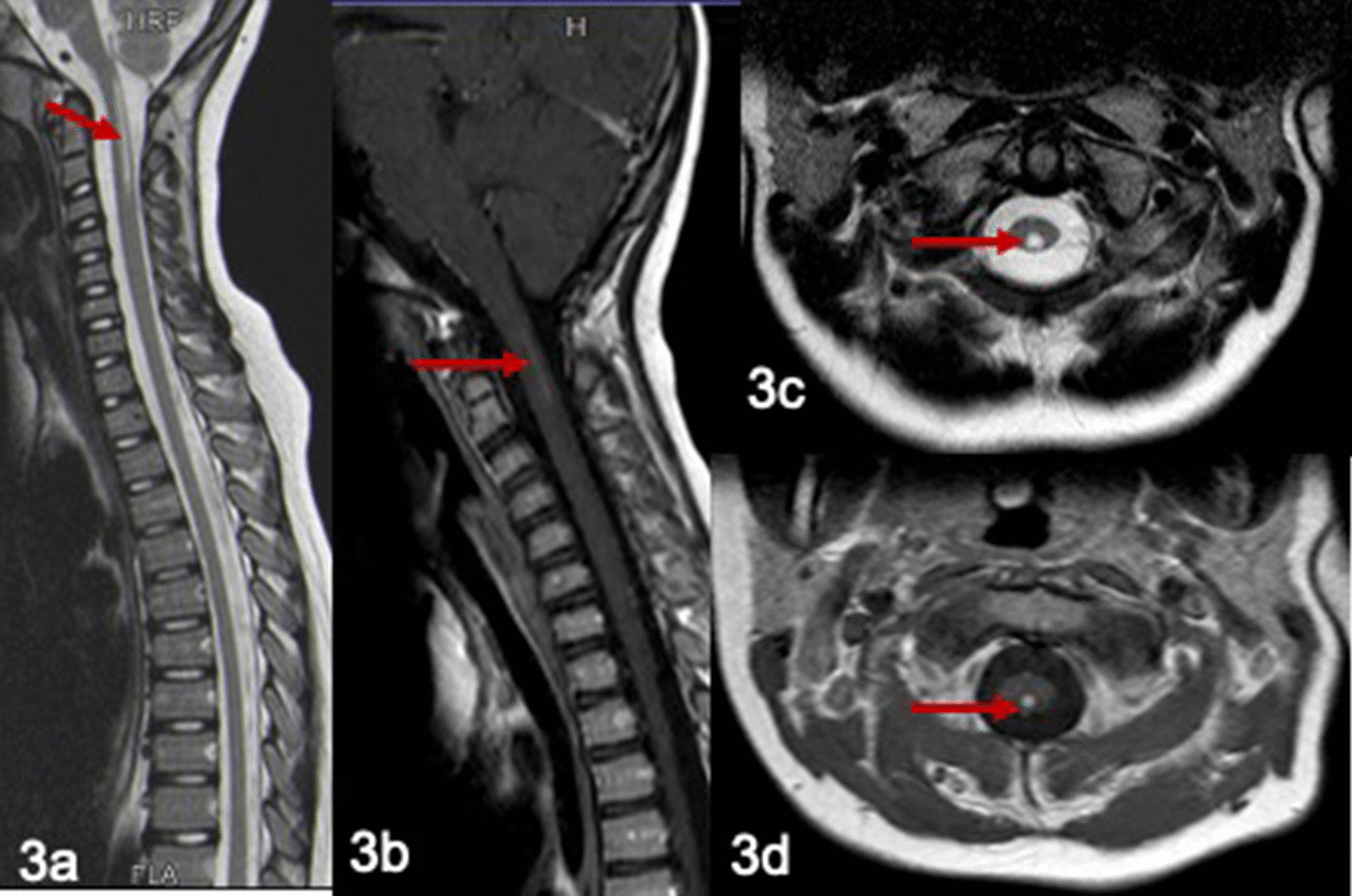
Spinal MRI in patient 2 at 9 years. T2 hyper-intense lesions in the posterior cervical cord extending from the level of the craniocervical junction to the C3 vertebral body (**a**, sagittal view, **c**, T2 weighted axial view). The lesions showed enhancement on administration of contrast (**b** sagittal, **d** axial view)

Spinal MRI at 9 years showed linear T2 hyper-intense lesions in the posterior cervical cord extending from the level of the craniocervical junction to the C3 vertebral body (Fig. 3a, T2 weighted sagittal image, Fig. 3c, T2 weighted axial view). The lesions showed enhancement on administration of contrast (Fig. 3b sagittal, Fig. 3d-axial view). Brain MRI at the same showed scattered focal areas of hyperintense signal in the frontal white matter The findings of repeated cranial and spinal MRI are essentially unchanged. The last MRI done at the age of 15 years did not show any white matter signal changes. MRI spine at the age of 17 years showed myelomalacia of the posterior cervical cord.

She presented with disabling neuropathic pain in the feet precipitated by intense exercise at the age of 16 years, responding to a short course of gabapentin. Repeat nerve conduction study showed persistence of motor axonal polyneuropathy in the lower limbs with mild worsening. She had two other self-limiting episodes of neuropathic pain in the feet until 19 years of age. MRI angiogram and venous doppler of lower limbs at age 17 years were normal.

During the last review, at the age of 19 years, she walks independently with ankle-foot orthoses and has normal intellect, participating in university undergraduate study. Examination showed mild temporal pallor of the optic disc, spasticity of the lower limbs with contractures of hamstrings and tendo-achilles. Sensory system examination showed normal touch and pain sensations but reduced position sense and vibration sense with a positive Romberg’s sign. There was mild discolouration of the toes and trophic changes of the nails. She had a spastic gait.

### Patient 3

She is the first child of consanguineous parents and is currently 56 years old. She was born at term after an uncomplicated pregnancy and delivery. The initial developmental milestones were normal. Gait difficulty was noted at the age of 3 years after an episode of febrile seizures. Since then she had progressive walking difficulty which deteriorated over the next 10 years, and was then static during her teenage years. She remained ambulant till the age of 11 years and needed assistance of a walking frame which progressed to wheel chair assistance during the third decade of life. During the course of the illness she received appropriate rehabilitative measures including orthopaedic procedures.

She has not had any unprovoked seizures. No learning difficulties were reported and there are no concerns with her vision. Neuropsychological profiling at the age of 47 demonstrated that she functions in the normal range intellectually. She has a psychiatric diagnosis of obsessive compulsive disorder that is currently well controlled on duloxetine.

On last follow up at the age of 56 years she was wheelchair dependent. Visual acuity was limited to finger counting close to the eyes. Fundi showed bilateral optic disc pallor. There was lower limb spasticity with bilateral extensor plantar responses. Lower limb lymphoedema was severe, and her extremities were cool and discoloured. She underwent an amputation of the right second and third metatarsals for chronic non-healing ulceration at age 50. Duplex ultrasound and venograms did not show vascular insufficiency. Deep tendon reflexes were brisk except ankle jerks which were bilaterally absent. Sensory system examination showed reduced sensation in both the feet, right foot more affected. There was impaired vibration and position sense. There were no cerebellar signs.

She underwent extensive neurometabolic investigations at presentation and the only finding was the persistent elevation in the plasma and CSF glycine levels which were to a lesser degree in comparison to her sister. She was diagnosed as variant non ketotic hyperglycinemia and was treated with protein restricted diet and sodium benzoate to lower her glycine levels which is being continued. Her plasma glycine levels were monitored throughout the course of the illness.

**Fig. 4:**
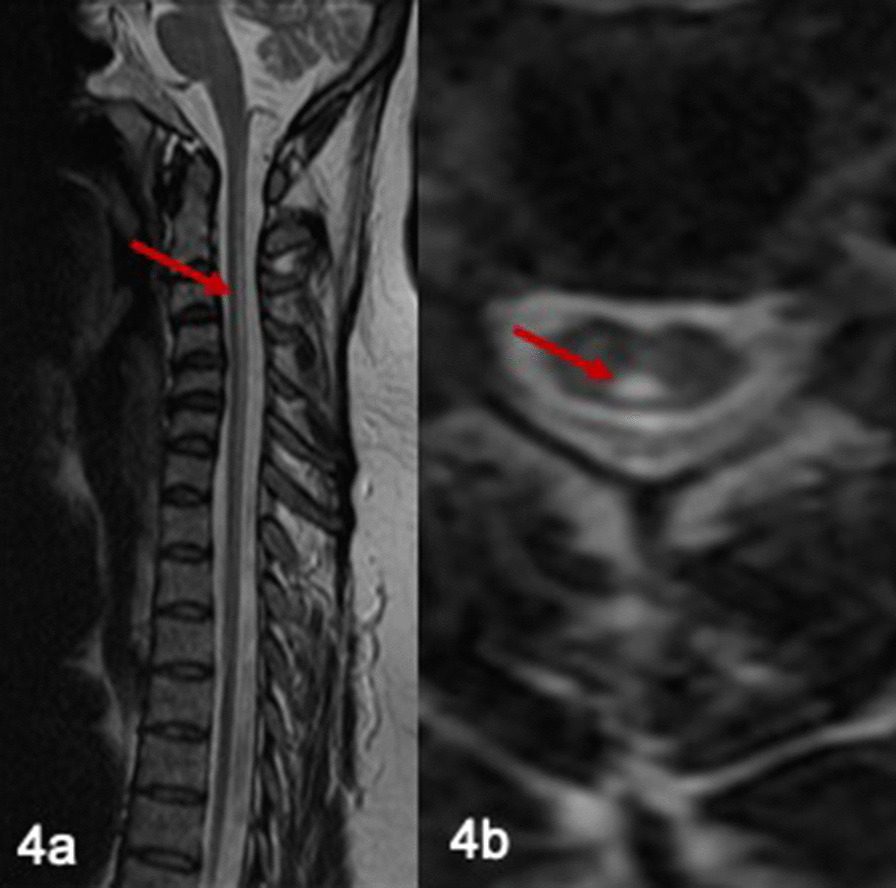
Spinal MRI in patient 3. MRI spine showed a focus of high signal in midline posteriorly extending from cranio-cervical junction to level T5-6 (**a**, **b**)

MRI brain performed at the age of 49 was normal. However, MRI spine showed a focus of high signal in midline posteriorly extending from cranio-cervical junction to level T5-6 (Fig. 4a, b).

### Patient 4

She is the younger sister of patient 3. She is currently 45 years years old and resides in a nursing home. She was initially seen at the age of six years due to concerns of frequent falls at school. However, she had gait difficulties for a long time before seeing a neurologist. On examination, she had spasticity of lower limbs with brisk deep tendon reflexes with extensor plantar response. She had a diplegic gait. Her gait progressively deteriorated but she remained independently ambulant until the age of 13 years. She could walk for 30-40 m with walking aids but requires wheelchair assistance most of the time. On follow up visits, there was increasing difficulty in obtaining her ankle jerks. Over the years, her assisted mobility has largely remained static, although she has deteriorated during intercurrent illnesses.

Her other problems include epilepsy, optic atrophy and learning difficulties. Her first seizure was at the age of 15 years, and optic atrophy (abnormal visual evoked potentials but normal electroretinogram) was detected a few years after the onset of epilepsy. The development of optic atrophy coincided with the use of vigabatrin, which was subsequently ceased. Both epilepsy and visual acuity have been stable during the last 10 years. She did not complete her schooling and her intellectual disability is mild.

**Fig. 5:**
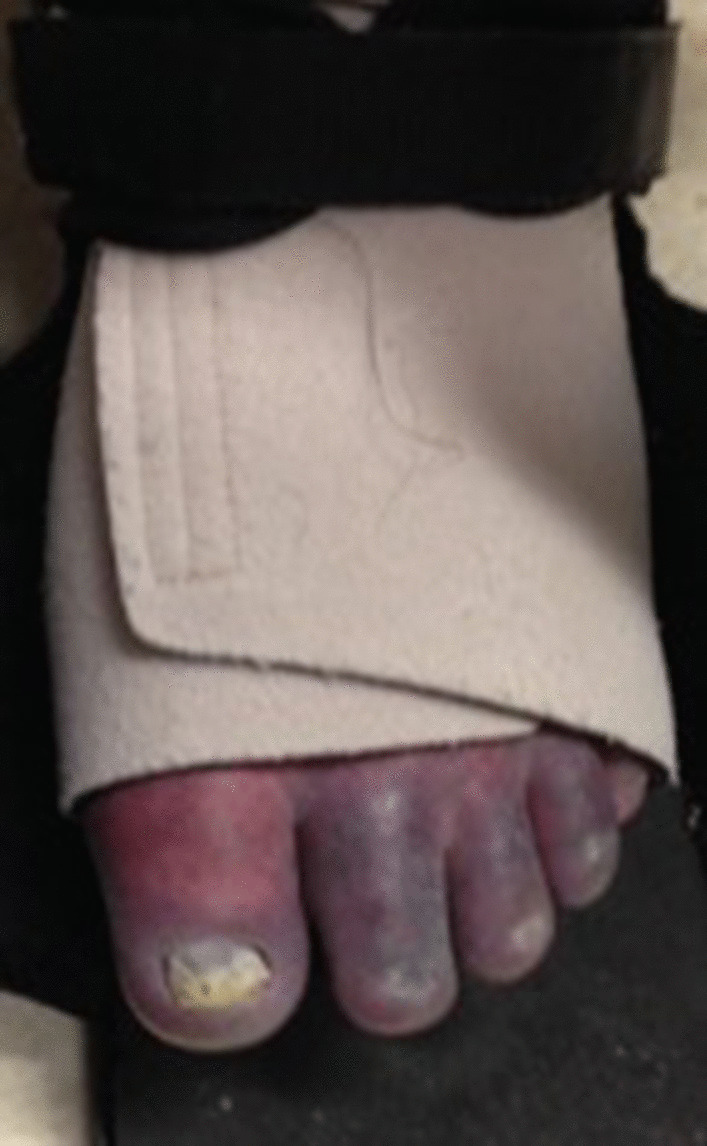
Photograph of feet showing discolouration of feet and trophic changes

On a recent examination at the age of 45 years, she was wheelchair dependent. There was profound oedema in both legs from below the knees. Both feet were discoloured with trophic changes (Fig. 5). She has not required lower limb digit amputation. She was alert and oriented to time, place and person. Her speech was clear and comprehensible. The upper limbs were normal except for weakness of the intrinsic hand muscles. In the lower limbs, there was spasticity with brisk knee jerks and bilateral extensor plantar response. Ankle jerks were absent bilaterally. Sensory system examination showed reduced touch, proprioception and vibration sense bilaterally. Mild postural intention tremors and mirroring movements were seen, but no dysmetria was observed.

**Fig. 6:**
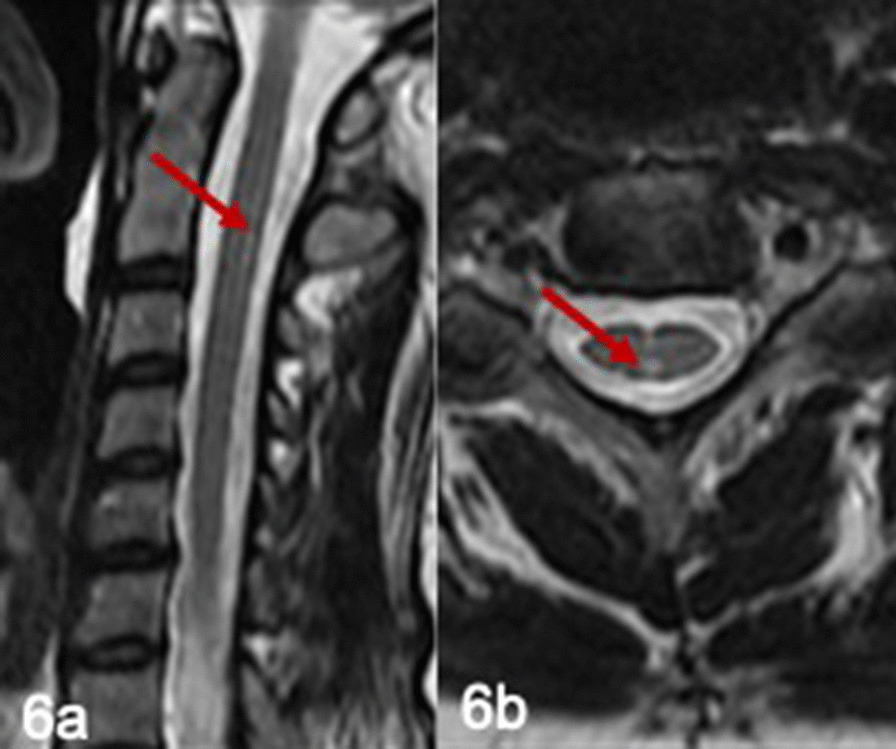
Spinal MRI in patient 4. T2 weighted sagittal (**a**) and axial view (**b**) shows a focus of high signal posteriorly in the cervical spine (arrows)

She underwent extensive neurometabolic investigations at presentation, which revealed raised plasma and CSF glycine with CSF: plasma glycine ratio of 0.04 (Table 1). A cranial CT was normal. MRI brain and spine performed at the age of 16 years were normal. MRI brain performed at the age of 38 years was normal and the MRI spine showed a focus of high signal in the midline posteriorly in the cervical spine (Fig. 6a, b). Nerve conduction studies showed absent compound muscle action potentials in the lower limbs with preserved sensory action potentials. Upper limbs showed normal conduction studies. There was evidence of denervation on needle electromyography. A repeat nerve conduction study showed absent sensory-motor and sensory action potentials in the lower limbs in keeping with severe polyneuropathy. Neuropsychological testing at the age of 39 was suggestive of borderline intellectual limitations. However, her visual impairment and limited educational opportunities make interpretation difficult.

## Discussion

Mitochondrial Fe-S cluster machinery defects have been implicated in several human diseases; collectively known as Fe-S cluster diseases [5, 6, 14]. In this report, we have characterised the clinical features, MRI findings, and long term follow up in four patients with GLRX5-associated [Fe-S] cluster disease, all caused by the same genetic mutation c.151_153delAAG (p.K51del). All had onset in early or late childhood with gait difficulty and preserved cognition suggestive of hereditary spastic paraplegia. The spastic paraparesis had a very gradual progression reaching moderate disability during adulthood, requiring orthopaedic intervention procedures for severe spasticity and contractures. Additional symptoms include optic atrophy, seizures, variable intellectual disability ranging from normal to mild, and a length-dependent motor/ sensory-motor axonal neuropathy. An additional patient reported in the literature manifested with spastic paraplegia and optic atrophy [10]. Recently two further cases have been published with both having onset in the second year of life. The Han Chinese case had compound heterozygote variants in GRXL5 with the first being the recognised c.151_153del and the second reported as variant of unknown significance (VUS)-c.196 C>T but predicted to confer a STOP codon [12]. This patient presented with encephalopathy after a viral illness, with elevations of glycine levels. She had recurrent episodes of encephalopathy but had persistent paraplegia until she died aged 4 years. The second patient aged 9 years from Turkey presented with a spastic gait at 15 months. He also had, elevated serum glycine, optic atrophy by 5 years and periventricular white matter abnormalities noted. Homozygous VUS c.347G > A is reported. Interestingly there are no reports of haematological manifestations especially sideroblastic anaemia in any of these, or our cases.

The disorder has been designated as spasticty, childhood early onset with hyperglycinemia OMIM #616859 ‘S–SPAHGC, highlighting the dominant phenotypic feature in these patients namely spastic paraplegia. lExtended follow up shows the clinical features are largely consistent with SPOAN (spastic paraplegia, optic atrophy and distal neuropathy, OMIM) or a restricted form of SPOAN. This has been described in another Fe-S cluster biogenesis disorder due to mutations in *IBA57*.^10^ It is generally believed that the defects involving early acting ISC components such as HSPA9 and GLRX5 lead to mitochondrial iron accumulation and sideroblasts. This is because of the role of these core ISC components in the maturation of cytosolic and nuclear Fe-S proteins, especially cytosolic aconitase. IRPI, the apo form of aconitase, also has a crucial role in cellular iron uptake regulation. In contrast, mutations in late acting mitochondrial ISC genes such as *ISCA1-ISCA2* and *IBA57* usually lead to multiple mitochondrial dysfunction syndrome and do not cause iron dysregulation because of their non-involvement in extramitochondrial Fe-S protein metabolism. Surprisingly, the neurological phenotype of GLRX5 mutation, comprising myelopathy and variable leukoencephalopathy, seizures, optic atrophy and peripheral neuropathy, is reminiscent of other multiple mitochondrial dysfunction syndromes caused by defects in late acting ISC proteins [6]. However mitochondrial respiratory enzymes levels in patients with *GLRX5* mutations have been reported to be normal when analysed in fibroblasts and patients who lacked lactic acidosis [8]. Nevertheless, it is noteworthy that one of our patients had a persistent lactate peak detected on MR spectroscopy suggesting indirect evidence for mitochondrial respiratory chain dysfunction.

In addition to our four cases and three others with compatible GRXL5 variants, there are potentially five other cases from the literature that have elevated glycine levels with findings within the phenotypic spectrum we describe. The first report was three adult brothers with spastic diplegia from Lebanon reported by Bank and Morrow [15]. A further case with similar findings specifically from the same village as our cases was reported by Steiman et al. [16]. A further Japanese case was reported with optic atrophy in adult life although motor findings are not described, potentially making 12 cases over all [17].

The consistent and predictable neurological phenotype as described above in patients with p.K51del *GLRX5* mutations is in stark contrast to the pyridoxine resistant sideroblastic anemia reported in association with missense mutations of *GLRX5 *[7, 11]. The pathophysiological basis of these two tissue-specific manifestations is intriguing, given the low tissue specificity of GLRX5 protein expression. The occurrence of pyridoxine resistant sideroblastic anemia is discernible given the role of GLRX5 in both mitochondrial and cytosolic Fe homeostasis [18]. The first reported case of sideroblastic anemia from GLRX5 deficiency was due to mutation in the first exon of the *GLRX5* gene that interferes with RNA splicing [7]. Experimental studies have shown that GLRX5 deficiency leads to impaired [Fe-S] assembly on iron regulatory protein (IRP1) and ferrochelatase (FECH) required for cellular iron homeostasis maintenance and heme biosynthesis, respectively [18]. Consequently, there was inhibited heme biosynthesis and cytosolic iron depletion. In a second patient who is a compound heterozygote (c.301 A > C and c.443 T > C), it has been demonstrated that the two *GLRX5* mutations impair [Fe-S] biogenesis in blood cells [11]. Extensive biochemical studies in three patients with p.K51del showed that the mutation resulted in Pyruvate Dehydrogenase (PDH) and α-Ketoglutarate Dehydrogenase (α-KGDH) deficiency and reduced lipoylation rather than GLRX5 deficiency [8]. The biosynthesis of lipoate requires lipoate synthase, which needs an [Fe-S] in an S-adenosylmethionine dependent reaction. Further studies in K562 cells have proven that GLRX5 protein has multiple roles in [Fe-S] protein synthesis and maturation [19]. The GLRX5null K562 cells and K562 cells expressing K101Q showed reduced PDH and α-KGDH enzyme activities, whereas those with L148S and L148S/K101Q mutants did not. This indicates that the L148S mutation leads to reduced Fe-S-IRP1, FE-S-m-aconitase and FE-S FECH levels; however, it does not affect lipoylation. The p.K51del mutation resulted in normal mitochondrial aconitase activity.

The MR imaging findings in these patients revealed very peculiar findings. Except in the first patient who had confluent white matter signal changes consistent with a mitochondrial leukoencephalopathy, all four of them had dorsal spinal cord signal changes with contrast enhancement in those it was administered. Two additional patients reported in the literature also had significant spinal cord signal changes, although one also had significant CNS disease [10, 12]. It is tempting to refer to these clinical and imaging findings as a ‘mitochondrial myelopathy’. This is also similar to the spinal cord signal changes reported in DARS associated leukoencephalopathy [20]. Similar to DARS-associated leukoencephalopathy, findings in *GLRX5* associated neurological disorder might confuse neurologists toward diagnosing an acquired inflammatory disorder. The contrast enhancement noted in one patient also highlights the interesting association of mitochondria and inflammation as reported previously and has been highlighted in recent reports [21, 22]. However, the persistent non-ketotic hyperglycinemia was an indication of a metabolic etiology. It remains to be seen whether anti-inflammatory treatment with steroids has a disease modifying effect. Steroids were used in patient one and changes persisted long-term. In patient 2 with less dramatic white matter changes in childhood, improvement occurred over years without anti-inflammatory treatment. In the two oldest siblings there was no cerebral white matter involvement when scanned in adulthood. Improving white matter lesions have also been described in FDX related Fe-S cluster biogenesis disorder [23].

The evolution of peripheral neuropathy in our patients is also noteworthy. Even though the length-dependent sensory-motor axonal neuropathy was largely subclinical during the initial years, it gave rise to disabling symptoms during adulthood. In particular, the disabling exercise-induced neuropathic pain in one patient and the combination of distal lower limb oedema, temperature instability, discolouration and pain akin to “reflex sympathetic dystrophy” in the siblings could be attributed to progressive small fibre involvement [24]. Leaky micro-vessels are one of the characteristic signs of small fibre damage and lead to oedema, sometimes blisters and abnormal colour and temperature. Denervation of the arteriovenous shunts allows direct blood flow from the arterioles to venules, bypassing the capillary beds resulting in skin engorgement while the deep tissues remain hypoxic [25]. Even though this has not been supported by skin biopsy to assess the nerve fibre density, this seems to be the most plausible explanation for the lower limb symptoms for these patients.

Biochemically all patients showed elevated plasma and CSF Glycine with a normal CSF/plasma Glycine ratio. This shows that even moderately elevated levels of plasma Glycine levels with a normal CSF/glycine ratio warrants further evaluation in the appropriate clinical context. It is possible that urine glycine elevation could screen for this disorder but we do not have those data for these patients prior to treatment with sodium benzoate. The clinical course in these patients brings out interesting therapeutic options which require further exploration. Patient 1 could not comply with sodium benzoate treatment, having significant persistent glycine elevations over childhood compared to the other three patients with control in standard target ranges for attenuated NKH (Fig. 1) [26]. Patient 2 had better clinical outcomes at a similar ages to patient 1 with significantly better control but other variables may have had an influence. Biochemically we were unable to define a role for lipoate in one patient that tried this medication. This has also been reported in patients with NFU1 mutations [27]. It is important to note that the lipoic acid moiety is integral to other enzyme complexes including pyruvate dehydrogenase, branched-chain ketodehydrogenase and alpha-keto-dehydrogenase complexes. These multiple levels of energy metabolism could lead to pathophysiology of the manifestations and may need to be accommodated in treatment [8].

Additional infrequent symptoms noted include seizures and psychiatric disturbances in the oldest patient. Seizures are an infrequent symptom and this could be expected from a lack of cortical involvement on MRI. The features were consistent with generalised genetic epilepsy syndrome. As expected from a disorder impacting mitochondrial function sodium valproate worsened the seizures in one of them, and the most effective drug was lamotrigine. Dextromethorphan has been tried without much benefit in this patient. Electroencephalogram on multiple occasions revealed normal findings. The psychiatric disturbances required the use of psychotropic drugs. It was also noted that the behavioural disturbances were less prominent when patients were on glycine lowering agents.

## Conclusions

We have characterized a distinct neurological phenotype associated with GLRX5 associated [Fe-S] cluster biogenesis disorder with all four patients being consistently managed by our paediatric and adult services between 10 and 25 years. The course has stabilized but is slowly progressive and may have been ameliorated using sodium benzoate. There are potential alternative metabolic pathways involved in disease pathogenesis but these require further study. It is hoped that a greater understanding of the Fe-S biogenesis may lead to the identification of a common way to circumvent Fe-S cluster biogenesis defects and mitochondrial iron overload.

## Data Availability

Data is stored on hospital database and can be accessed with appropriate consent processes.
